# Prospective client survey and participatory process ahead of opening a mobile drug consumption room in Lisbon

**DOI:** 10.1186/s12954-019-0319-1

**Published:** 2019-08-09

**Authors:** Hannah Taylor, Adriana Curado, Joana Tavares, Miguel Oliveira, Diana Gautier, João Santa Maria

**Affiliations:** 1 Universidade Nova de Lisboa Faculdade de Ciencias Sociais e Humanas, Lisbon, Portugal; 2 GAT - Grupo de Ativistas em Tratamentos, Lisbon, Portugal; 3 Médicos do Mundo, Lisbon, Portugal

**Keywords:** Drug consumption rooms, Mobile drug consumption room, Prospective client survey, People who inject drugs, Harm reduction, Portugal

## Abstract

**Background:**

Ahead of opening Portugal’s first mobile drug consumption room (MDCR) in Lisbon, information from People Who Use Drugs (PWUD) and local community members was necessary to determine current needs and shape the intervention. A participatory and peer-led process was ensured at all stages of data gathering and planning of the intervention.

**Methods:**

Prospective clients were surveyed to determine their willingness to use the service and preferences for use and to gain sociodemographic information. Persons over the age of 18 who reported injection drug use (PWID) were recruited using convenience sampling in the main open drug use scenes in Lisbon. In-person interviews were conducted by trained peer workers between November and December of 2017. The results (*n* = 72) of the questionnaires were analyzed, providing descriptive statistics.

**Results:**

There is a high level of willingness to use the MDCR, primarily for reasons of hygiene, privacy, and security. Most participants expressed a desire to use the MDCR daily. Potential clients are socially marginalized, and many suffer from unstable housing. Most are daily users and engage in unsafe injecting practices, such as public injecting and material sharing. High levels of hepatitis C, HIV, and hepatitis B were observed among the target population with low levels of healthcare access and utilization. Preferences were gauged regarding the scheduling of the MDCR’s hours and amount of time willing to travel to reach the MDCR and will be taken into account for implementation. The combination of high levels of willingness to utilize the service and high levels of need among the target population support the implementation of Lisbon’s first MDCR.

**Conclusions:**

Continual participation of PWUD and other community members will be necessary to maximize the public health and social impacts of this intervention, relative to this baseline. The plan to continue the participatory and peer-led development of the MDCR includes integrating peer workers, clients, and local community members within the operation, management, and evaluation of the service. This research adds to a growing literature about drug consumption rooms (DCRs) in Europe, which is especially limited concerning MDCRs.

## Background

Drug consumption rooms (DCRs) are both public health and social interventions and aim to prevent disease transmission and overdose-related deaths [4, 18] as well as to mitigate the negative community-level effects of public drug use [1]. In addition to providing direct services, DCRs offer education on safer injecting practices [23]. Users tend to adopt these safer practices even outside of the facility [6]. Furthermore, DCRs act as key sites for a referral to further services [13, 23] for people who use drugs (PWUD).

DCRs attract the most socially marginalized [23] and at-risk users in terms of health [12, 17, 23], and therefore represent harm reduction mechanisms with a great potential for impact. Users of DCRs are more likely to be in unstable housing situations [21], have recently overdosed [21], and be at elevated risk of bloodborne diseases from unsafe injection or syringe-sharing practices [21].

Positive health outcomes of DCR utilization include fewer overdoses and overdose deaths [12]. In addition, the interplay between behavioral changes and health outcomes include less rushed injecting [17], syringe sharing [23], and HIV risk behavior [12]. Positive effects for the surrounding community expand to less public injecting, less unsafe syringe disposal [17, 18], and less public disorder [23]. While most studies did not find increased drug dealing, trafficking, or crime in the areas surrounding the DCR [12, 18, 23], limited evidence has demonstrated increased aggressive incidents in the vicinity of the site, petty crime increases, and resentment on the part of local residents [12].

DCRs have been legally possible in Portugal for almost two decades but have not before been implemented. The overhaul of Portugal’s drug policy is summarized in the 1999 National Drug Strategy, which lays out a shift toward a less repressive drug policy and one centered on humanism, pragmatism, and public health. In 2001, both the decriminalization of low-level possession and use of illicit drugs and the Decree-law 183, regulating harm reduction responses, came into effect. DCRs are among the harm reduction measures detailed. However, the relevant law restricts DCR locations to areas that are not densely populated. The opening of a MDCR, which is part of a larger initiative by the city government and harm reduction NGOs to open 3 DCRs, two fixed and one mobile, allows service of the densely populated urban center. Only injection consumption is possible in the van due to limited space and lack of smoke extraction capacities.

DCRs are developed with their target population in consideration and therefore customized to the local drug-using population [1]. In order to maximize the benefits for users of DCRs, feasibility studies are utilized to understand the demand for DCRs [1, 2, 7, 10, 14] and characteristics of users ahead of opening DCRs [11] and call on both PWUD and other local experts.

Generally, the level of willingness to utilize a consumption site is high [1, 11]. Prospective surveys also assess the concerns of the PWUD population concerning the consumption site [1, 11], such as deterrents for use. Furthermore, they provide information on the anticipated frequency of use and desired scheduling [14] and location [10]. Finally, these surveys help the implementers of the DCRs ensure that they will reach the most vulnerable population [2, 7, 10, 11, 14]. Together, this information allows those in charge of implementing the site to maximize its reach and efficacy.

Once the service is implemented, there is a need for continual evaluation of the service’s efficacy and user satisfaction [16, 17]. In addition to the constraints that DCRs face, such as operating hours and waiting times [17], there are additional challenges of capacity, weather conditions, and other location-specific limitations unique to MCDRs [16].

The implementation of a pilot MDCR is a collaboration between Grupo de Ativistas em Tratamentos (GAT), Médicos do Mundo, and the City Council of Lisbon. Activities carried out through September of 2017 to January 2018 were divided into three main categories: diagnostic, training for teams and partners, and preparation of a final report. The diagnostic process included defining the methodology and data collection instruments for the site, field work including identification and characterization of intervention areas and conceptualization of consumption spaces, local partner meetings, and a prospective client survey. This survey of prospective clients provided data on socioeconomic characteristics, consumption patterns, health status, and both acceptability of and preferences for a MDCR in Lisbon.

Within the field of harm reduction, there is an ever-growing commitment to integrate peers in all aspects of research and intervention [8, 15, 20]. Peers offer unique skills and knowledge in the harm reduction field because of their lived experiences [15]. One broad definition of peers is that they are “any persons with equal standing within a particular community who share a common lived experience” [20]. For the purpose of this study, peers are persons who have used or currently use drugs. Additionally, they comprise a subset of PWUD because they are PWUD who are contributing to the harm reduction intervention in question. Peer workers are peers who are trained to administer harm reduction services and are paid staff members.

In the planning of this intervention, care has been taken to ensure the participation of peers. Peer workers administered the survey to users and interfaced with community members during the fieldwork period. Additionally, Consumidores Associados Sobrevivem Organizados (CASO), the only association of people who use drugs in Portugal, has participated since the beginning of the discussion concerning DCRs in Lisbon. The recommendations made by CASO for the implementation of DCRs can be found in the Appendix. The plan to continue a participatory and peer-led process for the DCR extends to the operation, management, and evaluation of the service. The results of the prospective client survey and how they, alongside a peer-led planning process, guide the intervention’s implementation are the primary focus of the present paper.

## Methods

A survey of prospective clients of the MDCR was carried out to learn about the target population, their needs, and their preferences for a potential mobile unit. The design of the survey was influenced both by literature reviews and the advice of peers, specifically peer workers who are GAT staff and from members of CASO who are not GAT staff. The survey was divided into three main sections: sociodemographic characteristics, patterns of consumption and use behaviors, and acceptability and willingness to use a MDCR in Lisbon.

Questionnaires were administered by trained peer workers between November 10 and December 18, 2017, during the outreach period in the communities identified as potential intervention sites. Persons over the age of 18 who reported current injection drug use were eligible to participate, as they comprise the target population of the intervention. Those reporting other types of consumption, namely smoking, were excluded from participating. There were no monetary incentives offered for participation. Informed consent was collected from all participants, including an agreement that their responses may be included in scientific publications. No additional ethical review was sought for this study. Interviews were conducted in Portuguese. Given the small sample size (*n* = 72), analysis of the results was limited to descriptive statistics.

## Results

### Sociodemographic characteristics of participants

Participants in the study were mostly men (81%), Portuguese (75%), and over 40 years old (69%), with a mean age of 43.53 years. Most of the participants born outside of Portugal come from African Portuguese Speaking Countries (72%). Forty percent of the participants were in unstable, versus stable, housing situations corresponding to the ETHOS [5] definition of homelessness at the time of survey administration. Among these, most (72%) sleep on the street or in a temporary shelter. See Table 6 in the Appendix for percentages of respondents by type of insecure housing (Table 1).

**Table 1:** Sociodemographic characteristics of participants (*n* = 72)

	Number	Percent
Gender		
Male	58	81
Female	13	18
Transgender	1	1
Age group		
< 40 years	22	31
> 40 years	50	69
Country of birth		
Portugal	54	75
Other	18	25
Housing situation		
Unstable	29	40
Stable	43	60

### Consumption patterns and associated behaviors

The vast majority of participants reported injected consumption of crack cocaine (79%) and heroin (75%) in the last 6 months. Other substances injected were powder cocaine (43%), benzodiazepines (15%), buprenorphine (6%), and methadone (3%).

Most participants are daily (57%) or regular users (25%), reporting at least one weekly consumption. Of those surveyed, 14% used syringes and/or needles they knew or suspected had been used by another person in the last 6 months, and 24% reported having shared other injection material such as spoons/cups, filters, water, or alcohol pads in the last 6 months. Regarding the site of consumption, 61% of respondents reported a public space (Table 2).

**Table 2:** Drug use patterns in the past 6 months (*n* = 72)

	Number	Percent
Frequency of injection drug use		
Every day	41	57
From time to time, not every week	13	18
Once or twice a week	8	11
Three to five times per week	10	14
Substances injected		
Crack cocaine	57	79
Heroin	54	75
Cocaine powder	31	43
Benzodiazepine	11	15
Buprenorphine	4	6
Methadone	2	3
Other	1	1
Sites used for consumption		
On the street	32	44
In own home or friend’s home	30	42
Open fields	19	26
In public bathrooms	13	18
In the car	9	13
In abandoned buildings	6	8
In occupied homes	4	6
In improvised, user-managed rooms	3	4
Other locations	2	3
In temporary lodges	1	1

When asked about opioid overdoses in the past 12 months, 8% of users (six users) reported having at least one overdose. Eighty-eight percent of all participants (*n* = 58) reported currently being in an opioid substitution program.

To better understand the current health statuses of participants, questions were asked regarding the most frequently transmitted infections among PWID (HIV, hepatitis B, and hepatitis C) and associated complications, such as vein damage and bacterial infections. Regarding HIV infection, 14% of the respondents reported positive serological status, of which 60% indicated that they were not on treatment (Table 3).

**Table 3:** Self-reported serostatus (*n* = 72)

	Number	Percent
HIV serostatus		
Negative	56	78
Positive	10	14
Do not know	5	7
Did not respond	1	1
HCV serostatus		
Negative	35	49
Positive	31	43
Do not know	5	7
Did not respond	1	1
HBV serostatus		
Negative	47	56
Positive	4	6
Do not know	20	28
Did not respond	1	1

Regarding hepatitis C Virus (HCV) infection, 43% reported positive serological status, of which only 3% indicated being in treatment, 23% on hospital follow-up, and the majority (74%) answered not being treated, nor on follow-up. Of the participants who reported negative serological status, 23% reported that they either were spontaneously cured or successfully treated. Hepatitis B virus (HBV) infection was less prevalent.

As for other complications associated with injected consumption, especially related to lack of hygiene and bad injection practices, 29% of the participants reported a bacterial infection in the last 6 months and 32% reported some type of vein damage in the last 6 months.

### Acceptability and willingness to use a mobile drug consumption unit in Lisbon

The final section of the survey was intended to access users’ acceptability and willingness to use a MDCR. Eighty-nine percent indicated that they were willing to use such a service. Among the 7% of participants who reported being unwilling to use the MDCR, the primary reason was already having a space to consume. Of those willing, all cited that they would use it due to hygiene reasons (100%), and the vast majority (89%) stated that they would do so for reasons of privacy and security (Table 4).

**Table 4:** Utilization potential for a MDCR in Lisbon

	Number	Percent
Willingness to use		
Yes	64	89
No	5	7
Do not know	3	4
Reasons for using a MDCR		
Security: fear of overdose, to avoid material sharing, avoiding street, or police violence	57	89
Privacy: to avoid having to consume in public spaces	57	89
Hygiene: to have access to a clean space for consumption and necessary equipment	64	100
Support from a specialized team	50	78
Access to other services such as nurses, screening, referrals, peer counseling	15	23
Other reasons	1	2
Reasons not to use a MDCR		
Already have a space to consume	4	80
Privacy: afraid of confidentiality breaches by the team, do not wish to be seen entering the space, do not wish to be seen by other clients	0	0
Prefer to consume alone	1	20
Worried about other clients’ behavior	1	20
Ashamed to consume in front of a technician	2	40
Frequency of use of the MDCR		
Every day	48	75
Several times per week	5	8
At least once per month	3	5
Once or twice	0	0
Do not know	8	13

For those who indicated willingness to use the MDCR, information was collected on how often they wish to use the service, their preferences regarding operating times, and how much time they were willing to travel to reach a mobile unit. The majority (75%) indicated a willingness to use the mobile unit every day. For time willing to travel, 27% reported being willing to travel between 21 and 30 min, while the majority of participants reported being willing to travel for a lesser amount of time (Table 5).

**Table 5:** Preferences for MDCR operation

	Number	Percent
Preferred operation hours for a MDCR		
Mornings (8 a.m.–12 p.m.)	14	22
Afternoon (12 p.m.–4 p.m.)	1	2
Evening (4 p.m.–8 p.m.)	17	27
Night (8 p.m.–12 a.m.)	13	20
Any time	18	28
Do not know	1	2
Time willing to travel to reach a MDCR		
1–5 min	5	8
6–10 min	16	25
11–20 min	18	28
21–30 min	17	27
Do not know	7	11
No information	1	2

## Discussion

With high levels of desire to participate and potential clients presenting high levels of health and social risk, Lisbon’s first MDCR holds the potential to improve the health, safety, and quality of life of PWID and communities affected by consumption in public spaces. Current harm reduction services available in Lisbon include mobile methadone units, street teams which provide material exchanges, treatment centers, rapid testing, and referrals within this network and to other health and social services. None of these services provides a safe space for users in a moment of heightened risk: that of consumption. Therefore, this initiative represents a complementary service to what already exists. The relatively small (23%) percentage of people seeking referrals does not necessarily indicate that users only need a safe place to consume, but rather that there is a strong network of harm reduction services available in Lisbon and that they are already accessing them, but that this service is still missing. Anticipated positive outcomes include access to safer injecting conditions, reducing morbidity and mortality associated with overdose and the harms associated with injecting, service access and referral of PWIDs to existing networks of health and social services Lisbon, and increased public awareness and acceptance concerning the benefits of DCRs. Expected positive community-level impacts include less public syringe disposal and less public injecting [17, 18].

MDCRs represent a newer addition to the harm reduction sphere in terms of injection facilities, with benefits being their flexibility to change location in accordance with the needs of their target population and to respond to drug market shifts or pressures from other sources such as law enforcement [3]. This represents a shift toward safer environment interventions [19] rather than pushing for social or behavioral change on the part of users. For MDCRs to successfully serve the populations that they operate within, it is necessary to have supportive local environments both in terms of local government and law enforcement and the community members surrounding the service [3]. Though mobile units tend to have lower capacity than fixed locations, they can be complementary to the services offered at fixed locations or open up doors for their implementation [3].

The team responsible for implementation is committed to an integrated and participatory approach to the health and social needs of PWID in Lisbon. All intervention is guided by the principles of public health, human rights, and evidence-based practices. In addition to integrating peers in the planning and evaluation processes, participation extends to professionals working in the area and local and strategic partners. In accordance with this, proposals for locations of the MDCR stops come from the insights of the neighborhood teams and local partners and prioritize access to users, privacy, and the needs of the local community. As the service operates, consistent communication with clients will be fostered in order to adapt to their needs and opinions.

Furthermore, in order to ensure that the service provision is in line with the needs of the target population and to give PWUD a voice in the program’s management, trained peer workers will be a part of the staff. Peers will also work in the proximity of the mobile unit to provide information, mediate conflicts, collect materials, and invite PWUD to engage with the initiative. This will minimize any negative impacts of the intervention on the public space and help foster a climate of acceptance.

Though DCRs operate in diverse settings, much of the English-language literature originates from isolated cases. Notably, the Insite center in Vancouver has been well-studied [6, 11, 21–23], as has a DCR in Sydney [13, 17]. In Europe [12], where the majority of DCRs operate, much of the literature is not in English [1, 9]. This paper also adds field knowledge concerning a specific and less-well-documented form of consumption space, a MDCR. Furthermore, the insights gained from the involvement of peers in all aspects of the implementation, from advising, to research, to publication and evaluation of the service, represent an important addition to the literature.

Limitations of the current study include a small sample size, which limits the statistical power and generalizability of the findings to the larger population of PWID in Portugal. However, this research provides a baseline of the characteristics, behaviors, and attitudes of potential clients. Future analyses of those who utilize the service will be more robust in terms of sample size. This paper is intended to be the first in a series which continually evaluate this intervention for its target population and the larger community. Analysis of the impact of this intervention will not be limited to the experiences of clients but will also account for the response in terms of public health and social impact in the surrounding communities.

## Conclusions

Diagnostic information shows that PWID are an aging population, mostly over 40 years of age, and suffering from poverty and social exclusion, especially as it relates to housing. The intervention will target PWID with greater vulnerability who are consuming publicly or in other risk conditions. Most are regular or daily users of crack cocaine and heroin. The percentage who report sharing material is still significant enough to cause concern and likely is related to the public conditions in which they consume. Health-wise, the population has high levels of hepatitis C, HIV, and hepatitis B prevalence with low levels of treatment of these infections. Sixty percent of people living with HIV are not in treatment and 74% with hepatitis C are not receiving specialized care. The data highlights a prevalence of other complications associated with injected consumption, which, when untreated, can result in serious or even irreversible damages.

Given the overwhelming willingness to use the MDCR, it is expected that there will be an improvement in health outcomes among the target population of this service. Even PWID who do not utilize the service may benefit indirectly from a reduction in drug-related infections among their peers. Involvement of local and strategic partners will continue to be prioritized given the innovative and experimental nature of the proposed intervention as an addition to Portugal’s harm reduction landscape. Above all, peer input is considered necessary to arrive at a service that is in and of the community it serves.

## Data Availability

The survey questionnaire and informed consent forms used in this article are available upon request. The dataset analyzed during the current study is not available. Participants were assured during the informed consent process that individual responses were confidential.

## Appendix

**Table 6:** Characterization of precarious housing

	Number	Percent
No roof: sleeping in the street or overnight shelter or temporary shelter for less than 3 months, forced to spend several hours a day in a public space.	21	72
Homeless: living in a shelter, refugee centers, prison, community therapy, a psychiatric hospital.	3	10
Unsafe housing: living temporarily with family or friends (not by choice), not having a lease (excluding squatting), subject to threat of eviction, living under threat of partner or family violence.	2	7
Inadequate housing: living in a mobile home, caravan, van, squatting, living in overcrowded housing.	3	10
Total	29	100

Recommendations made by CASO for the implementation of the assisted consumption rooms:

1. Involvement of civil society is very important, especially of those affected, who possess unique experiential knowledge, easy access to the places where drugs are consumed, and privileged relationships of trust. With proper motivation and training, they can develop methods of proven effectiveness and efficiency, and at the same time that direct investment is channeled to the people who need it the most. We are promoting health literacy and rights, therefore, to enable active citizenship.
2. Adopt a model that is truly person-centered and pragmatic and therefore not hospital-based nor centered on disease and treatment.
3. Promote a model of governance that is as horizontal and participatory as possible (involving the local community, drug users, security forces, etc.).
4. The law restricting use within DCRs to injection should be updated.
5. Fixed-location DCRs should present a broad set of responses to the needs of those who use them (legal, medical, psychosocial and peer support, but also distribution of consumption material, clothing, personal hygiene, lockers, among others).
6. Mobile units should develop actions not only directed toward the interior of the van but should rather use the van as a focal point for interventions by teams that include peer educators, mediators, and consultants.

## Abbreviations

DCR: Drug consumption room
MDCR: Mobile drug consumption unit
PWID: People who inject drugs
PWUD: People who use drugs

## References

[1] Atkin-Brenninkmeyer E, Larkan F, Comiskey C (2017). Factors concerning access to a potential drug consumption room in Dublin, Ireland. Cogent Social Sciences.

[2] Broadhead RS, Borch CA, Van Hulst Y, Farrell J, Villemez WJ, Altice FL (2003). Safer injection sites in New York City: A utilization survey of injection drug users. J Drug Iss.

[3] Dietze P, Winter R, Pedrana A, Leicht A, Majo I Roca X, Brugal MT (2012). Mobile safe injecting facilities in Barcelona and Berlin. Int J Drug Policy.

[4] European Monitoring Centre for Drugs and Drug Addiction. Drug consumption rooms: an overview of provision and evidence. 2018. Available online: http://www.emcdda.europa.eu/system/files/publications/2734/POD_Drug%20consumption%20rooms.pdf. Accessed 12 Nov 2017.

[5] FEANTSA (2005). ETHOS–European typology on homelessness and housing exclusion..

[6] Fast D, Small W, Wood E, Kerr T (2008). The perspectives of injection drug users regarding safer injecting education delivered through a supervised injecting facility. Harm Reduct J.

[7] Green TC, Hankins CA, Palmer D, Boivin J, Platt R. My place, your place, or a safer place: the intention among Montreal injecting drug users to use supervised injecting facilities. Can J Public Health. 2004:110–4. 10.1007/BF03405777 PMC6975653 15074900

[8] Greer AM, Luchenski SA, Amlani AA, Lacroix K, Burmeister C, Buxton JA (2016). Peer engagement in harm reduction strategies and services: a critical case study and evaluation framework from British Columbia, Canada. BMC Public Health.

[9] Hedrich D, Kerr T, Dubois-Arber F. Drug consumption facilities in Europe and beyond. European Monitoring Centre for Drugs and Drug Addiction (EMCDDA), Harm reduction: evidence, impacts and challenges, Rhodes, T.and Hedrich, D.(eds), Scientific Monograph Series 2010;10.

[10] Hunt N, Lloyd C, Kimber J, Tompkins C (2007). Public injecting and willingness to use a drug consumption room among needle exchange programme attendees in the UK. Int J Drug Policy.

[11] Kerr T, Wood E, Small D, Palepu A, Tyndall MW (2003). Potential use of safer injecting facilities among injection drug users in Vancouver’s Downtown Eastside. CMAJ.

[12] Kimber J, Dolan K, Wodak A (2005). Survey of drug consumption rooms: service delivery and perceived public health and amenity impact. Drug Alcohol Rev.

[13] Kimber J, Mattick RP, Kaldor J, van Bleek I, Gilmour S, Rance JA (2008). Process and predictors of drug treatment referral and referral uptake at the Sydney Medically Supervised Injecting Centre. Drug Alcohol Rev.

[14] Kral AH, Wenger L, Carpenter L, Wood E, Kerr T, Bourgois P (2010). Acceptability of a safer injection facility among injection drug users in San Francisco. Drug Alcohol Depend.

[15] Marshall Z, Dechman M, Minichiello A, Alcock L, Harris GE (2015). Peering into the literature: a systematic review of the roles of people who inject drugs in harm reduction initiatives. Drug Alcohol Depend.

[16] Mema SC, Frosst G, Bridgeman J, Drake H, Dolman C, Lappalainen L (2019). Mobile supervised consumption services in Rural British Columbia: lessons learned. Harm Reduct J.

[17] Petrar S, Kerr T, Tyndall MW, Zhang R, Montaner JS, Wood E (2007). Injection drug users’ perceptions regarding use of a medically supervised safer injecting facility. Addict Behav.

[18] Potier C, Laprévote V, Dubois-Arber F, Cottencin O, Rolland B (2014). Supervised injection services: what has been demonstrated? A systematic literature review. Drug Alcohol Depend.

[19] Rhodes T, Kimber J, Small W, Fitzgerald J, Kerr T, Hickman M (2006). Public injecting and the need for ‘safer environment interventions’ in the reduction of drug-related harm. Addiction.

[20] Ti L, Tzemis D, Buxton JA (2012). Engaging people who use drugs in policy and program development: a review of the literature. Subst Abuse Treat Prev Policy.

[21] Wood E, Tyndall MW, Li K, Lloyd-Smith E, Small W, Montaner JS (2005). Do supervised injecting facilities attract higher-risk injection drug users?. Am J Prev Med.

[22] Wood E, Kerr T, Small W, Li K, Marsh DC, Montaner JS (2004). Changes in public order after the opening of a medically supervised safer injecting facility for illicit injection drug users. CMAJ.

[23] Wood E, Tyndall MW, Montaner JS, Kerr T (2006). Summary of findings from the evaluation of a pilot medically supervised safer injecting facility. CMAJ.

